# Dimensão latente de comorbidades e depressão em idosos: estudo transversal com dados da Pesquisa Nacional de Saúde, Brasil, 2019

**DOI:** 10.1590/S2237-96222026v35e20250722.pt

**Published:** 2026-10-12

**Authors:** Fabian Leonardo Muñoz, Renato Ferreira da Cruz, Liciana Vaz de Arruda Silveira, Maria del Pilar Diaz

**Affiliations:** 1 Universidad Nacional de Córdoba, Facultad de Ciencias Médicas, Córdoba, Argentina; 2 Universidade Federal de Mato Grosso, Barra do Garças, MT, Brasil; 3 Universidade Estadual Paulista, Instituto de Biociências, Botucatu, SP, Brasil

**Keywords:** Depressão, Comorbidade, Idoso, Fatores Socioeconômicos, Estudos Transversais, Depresión, Comorbilidad, Anciano, Factores Socioeconómicos, Estudios Transversales

## Abstract

**Objetivo:**

Construir uma dimensão latente de comorbidades e avaliar sua associação com a depressão em idosos brasileiros.

**Métodos:**

Estudo transversal com dados da Pesquisa Nacional de Saúde no Brasil de 2019, incluindo indivíduos com 60 anos ou mais. A dimensão latente de comorbidades foi construída com base em cinco condições autorreferidas: hipertensão arterial, diabetes mellitus, colesterol alto, doença arterial coronariana e acidente vascular cerebral. Utilizou-se a modelagem por meio de equações estruturais generalizadas (*Generalized Structural Equation Modeling*, GSEM). As razões de prevalência (RP) e os intervalos de confiança de 95% (IC95%) foram estimados por meio de regressão de Poisson com variância robusta.

**Resultados:**

Foram analisados 22.705 idosos, com prevalência de depressão de 11,8%. A hipertensão arterial foi a condição mais frequente (56,4%). A dimensão latente de comorbidades apresentou forte associação positiva com a depressão (RP 12,61; IC95% 11,59; 12,65). A prevalência do desfecho foi maior no sexo feminino (RP 2,82; IC95% 2,55; 3,12) e entre os de maior renda (RP 1,11 a 1,16), enquanto o avanço da idade mostrou-se inversamente associado à depressão (RP 0,98; IC95% 0,97; 0,99).

**Conclusão:**

A depressão em idosos brasileiros associou-se significativamente à carga de comorbidades, ao sexo feminino, à idade e à renda. A modelagem por dimensões latentes contribuiu para uma compreensão integrada das condições crônicas, revelando que o acúmulo dessas patologias exerce impacto substancial na saúde mental dessa população, reforçando a necessidade de abordagens multiprofissionais e integradas no cuidado ao idoso no Sistema Único de Saúde (SUS).

## Resume

**Objetivo:**

Construir una dimensión latente de comorbilidades y evaluar su asociación con la depresión en adultos mayores brasileños.

**Métodos:**

Estudio transversal que utilizó datos de la Encuesta Nacional de Salud de Brasil de 2019, incluyendo a personas de 60 años o más. La dimensión latente de comorbilidades se construyó a partir de cinco afecciones autodeclaradas: hipertensión arterial, diabetes mellitus, colesterol alto, enfermedad arterial coronaria y accidente cerebrovascular. Se empleó el modelado de ecuaciones estructurales generalizadas (GSEM). Se estimaron razones de prevalencia (RP) e intervalos de confianza del 95% (IC95%) mediante regresión de Poisson con varianza robusta.

**Resultados:**

Se analizó un total de 22.705 adultos mayores, con una prevalencia de depresión del 11,8%. La hipertensión arterial fue la afección más frecuente (56,4%). La dimensión latente de comorbilidades mostró una fuerte asociación positiva con la depresión (RP 12,61; IC95% 11,59; 12,65). La prevalencia del resultado fue mayor entre las mujeres (RP 2,82; IC95% 2,55; 3,12) y aquellos con mayores ingresos (RP 1,11 a 1,16), mientras que el aumento de la edad se asoció inversamente con la depresión (RP 0,98; IC95%: 0,97; 0,99).

**Conclusión:**

La depresión entre los brasileños de edad avanzada se asoció significativamente con la carga de comorbilidad, el sexo femenino, la edad y los ingresos. El modelado de dimensiones latentes contribuyó a una comprensión integrada de las enfermedades crónicas, revelando que la acumulación de estas patologías tiene un impacto sustancial en la salud mental de esta población, lo que refuerza la necesidad de enfoques multiprofesionales e integrados para la atención de las personas mayores en el Sistema Único de Salud de Brasil.

## Introdução

A depressão é um importante problema de saúde pública global; contribui para a perda de funcionalidade e para o aumento da carga de doenças, especialmente entre pessoas idosas. Em 2025, estimou-se que aproximadamente 332 milhões de pessoas no mundo convivem com a condição, o que a coloca entre os transtornos mentais mais comuns [1,2]. No Brasil, é um dos transtornos mentais mais comuns e recorrentes, com impacto negativo na qualidade de vida e aumento do risco de mortalidade [3,4]. 

Na velhice, a depressão destaca-se como um dos transtornos mentais mais frequentes, caracterizada por humor persistentemente rebaixado, apatia, redução de energia, alterações do sono e do peso corporal, além de prejuízos cognitivos, como dificuldade de concentração e lentificação psicomotora. Podem, ainda, ocorrer diminuição da libido, sentimento de culpa, pensamentos negativos e, em casos graves, ideação suicida [5]. Trata-se de uma condição de evolução geralmente prolongada, influenciada por múltiplos determinantes, incluindo componentes biológicos, psicológicos, sociais e ambientais [6]. Embora sua etiologia não esteja completamente elucidada, há consenso quanto à contribuição de fatores psicossociais e biológicos, bem como à associação com outros diagnósticos psiquiátricos, como transtornos de ansiedade e de personalidade [7]. Em idosos, o quadro tende a ser mais complexo, frequentemente decorrente da interação entre vulnerabilidades acumuladas ao longo da vida e desafios próprios dessa faixa etária [8]. 

A prevalência de depressão é maior no sexo feminino [3,9], estando associada ao avanço da idade, à menor escolaridade, à baixa renda, à solidão ou ao fato de viver sozinho, bem como à presença de doenças crônicas ou de mau estado de saúde, entre outros fatores [10]. 

No Brasil, o envelhecimento populacional tem ocorrido de forma acelerada. O número de pessoas com 60 anos ou mais aumentou de 20,6 milhões em 2010 para 32,1 milhões em 2022, o que representa um crescimento de 56% em pouco mais de uma década [11]. Avanços na medicina reduziram a mortalidade e aumentaram a expectativa de vida, mas elevaram a carga de doenças crônicas não transmissíveis, como hipertensão arterial, diabetes e doenças cardiovasculares, que coexistem e prejudicam a saúde mental [12]. O novo cenário demográfico é relevante para definir direitos e benefícios aos idosos. Deve-se reconhecer as necessidades específicas e os desafios do envelhecimento, como multimorbidades, limitações funcionais, baixa renda, abandono e violência [13,14].

No Brasil, em 2019, registrou-se prevalência de 13% de sintomas depressivos clinicamente relevantes entre idosos brasileiros [9]. Esses dados revelam variações significativas segundo sexo, idade e condições socioeconômicas, evidenciando maior carga da doença em mulheres e em indivíduos com menor escolaridade [9,15]. Por outro lado, em 2022, houve menor prevalência de depressão entre idosos residentes em áreas rurais e em regiões do Nordeste e do Norte do Brasil [16]. Tais diferenças geográficas podem refletir particularidades socioculturais, formas de organização familiar, redes de apoio locais e distintas percepções sobre saúde e adoecimento, que influenciam a expressão e o reconhecimento dos sintomas depressivos [17]. 

A coexistência de múltiplas doenças crônicas em idosos impõe limitações às análises baseadas em variáveis isoladas, uma vez que essas condições tendem a ocorrer de forma inter-relacionada. Para lidar com essa complexidade, empregou-se o Modelo de Equações Estruturais Generalizadas (*Generalized Structural Equation Modeling*, GSEM), abordagem que permite integrar, em uma única estrutura analítica, um componente de mensuração e um componente estrutural, possibilitando a construção de uma dimensão latente que sintetiza a carga global de comorbidades. O estudo teve como objetivo construir uma dimensão latente de comorbidades e avaliar sua associação com a depressão em idosos brasileiros.

## Métodos

### Delineamento

Trata-se de um estudo transversal de base populacional, com delineamento analítico, conduzido com base em dados secundários da Pesquisa Nacional de Saúde (PNS) de 2019. O estudo foi elaborado conforme as diretrizes do instrumento Fortalecimento do Relato de Estudos Observacionais em Epidemiologia (*Strengthening the Reporting of Observational Studies in Epidemiology*, STROBE).

### Contexto

A PNS é um inquérito domiciliar de abrangência nacional, conduzido pelo Instituto Brasileiro de Geografia e Estatística (IBGE), em parceria com o Ministério da Saúde. Para este estudo, utilizou-se a coleta de dados realizada em 2019 [18]. A pesquisa faz parte do Sistema Integrado de Pesquisas Domiciliares do IBGE e tem como objetivo produzir estimativas robustas sobre múltiplas dimensões das condições de saúde da população brasileira. Seu escopo temático abrange condições de saúde autorreferidas, morbidades crônicas, comportamentos relacionados ao estilo de vida, utilização de serviços de saúde e ações de prevenção e promoção da saúde. A cobertura geográfica é nacional, com representatividade em grandes regiões, Unidades da Federação, regiões metropolitanas e capitais [18].

### Participantes

A população-alvo do presente estudo foi composta de indivíduos com 60 anos ou mais de idade, residentes em domicílios particulares em todo o território nacional. Foram incluídos os indivíduos com informações completas sobre as variáveis de desfecho (depressão) e de exposição (condições crônicas investigadas).

A amostra da PNS 2019 foi selecionada por meio de um plano de amostragem probabilístico por conglomerados em três estágios. No primeiro estágio, foram selecionados setores censitários ou conjuntos de setores (unidades primárias de amostragem); no segundo, domicílios particulares permanentes nesses setores; e no terceiro, um morador adulto foi selecionado aleatoriamente em cada domicílio para responder ao questionário individual.

Para este estudo, foram considerados apenas os indivíduos com 60 anos ou mais que participaram do módulo individual. As entrevistas foram realizadas por pessoas previamente treinadas que utilizaram questionários padronizados [18].

### Fontes de dados

Os dados foram extraídos do banco de dados público da PNS 2019, disponível em https://www.pns.icict.fiocruz.br/. As variáveis sociodemográficas foram obtidas nos módulos C (Características gerais dos moradores), D (Características de educação) e F (Rendimentos domiciliares). As condições crônicas e a depressão foram obtidas no módulo Q (Doenças crônicas), com base em diagnósticos médicos prévios autorreferidos. Todas as informações foram coletadas por meio de autorrelatos em entrevistas presenciais.

### Variáveis

A variável desfecho foi a depressão, definida por diagnóstico autorreferido, previamente estabelecido por médico ou profissional de saúde mental (psiquiatra ou psicólogo), obtido com a pergunta: “Algum médico ou profissional de saúde mental já lhe deu o diagnóstico de depressão?”. Considerou-se a presença do desfecho quando a resposta foi “sim”.

A variável de exposição principal foi a dimensão latente de comorbidades, operacionalizada por meio de um GSEM, construída com base em cinco condições crônicas autorreferidas: hipertensão arterial (sim; não), diabetes (sim; não), colesterol alto (sim; não), doença arterial coronariana, incluindo infarto agudo do miocárdio ou angina (sim; não) e acidente vascular cerebral (sim; não). A variável latente foi especificada com a hipertensão arterial como indicador de referência (carga=1,00), o que nos permitiu estimar livremente os coeficientes das demais condições, a fim de identificar a contribuição diferencial de cada morbidade. As variáveis explicativas incluíram características sociodemográficas: sexo (masculino; feminino), faixa etária (60-64; 65-69; 70-74; ≥75 anos), escolaridade (primário; secundário; superior), renda domiciliar (0-2; 3-4; ≥5 salários mínimos) e número de co-residentes (1-3; 4-6; ≥7 moradores no domicílio). A categorização das variáveis baseou-se em abordagens amplamente utilizadas em estudos epidemiológicos, com o objetivo de captar diferentes níveis de risco e assegurar a robustez das estimativas (Tabela suplementar 1).

### Viés

Reconhece-se o viés de informação inerente ao autorrelato, que pode subestimar as prevalências entre indivíduos com menor acesso aos serviços de saúde. O delineamento transversal impede o estabelecimento de precedência temporal, o que configura risco de causalidade reversa. Também são plausíveis o viés de sobrevivência e o confundimento residual decorrente de variáveis não mensuradas. Para reduzir o viés de seleção, todas as análises incorporaram os fatores de expansão e o desenho amostral complexo da PNS.

### Tamanho do estudo

O tamanho da amostra não foi delimitado por cálculo específico para este estudo, visto que se utilizou a totalidade dos participantes elegíveis da PNS 2019 com 60 anos ou mais e com informações completas para todas as variáveis de interesse. No total foram 22.705 indivíduos.

### Métodos estatísticos

Inicialmente, realizou-se a análise descritiva, com distribuições de frequência absoluta e relativa, estimativas pontuais e intervalos de confiança de 95% (IC95%). As diferenças nas proporções entre indivíduos com e sem depressão foram avaliadas pelo teste do qui-quadrado, adotando-se o nível de significância de 5%.

A variável de exposição principal foi operacionalizada como a dimensão latente de comorbidades, estruturada no âmbito de um GSEM em dois componentes simultâneos: o de mensuração, para construir a dimensão latente, e o estrutural, para estimar a associação com a depressão, ajustando-se pelas covariáveis sociodemográficas. Os efeitos foram estimados por máxima verossimilhança. A dimensão latente de comorbidades é representada por uma elipse, e as variáveis observadas, por retângulos, com setas indicando os efeitos lineares (γ_n_) entre si. 

Para obter as razões de prevalência (RP) e os respectivos IC95%, aplicou-se a regressão de Poisson com variância robusta [19], indicada para estudos transversais com desfechos binários. Além do modelo geral, realizaram-se análises estratificadas por sexo e calcularam-se as razões de prevalência marginais preditivas por idade. A seleção final do modelo baseou-se nos critérios de informação de Akaike e Bayesiano, priorizando-se o melhor ajuste e a coerência teórica [20]. Casos com informações ausentes foram excluídos das análises específicas, sem imputação de dados.

As análises foram realizadas com o uso do software Data Analysis and Statistical (Stata), versão 17.0, utilizando o módulo *survey* (conjunto de comandos do programa estatístico destinado à análise de dados provenientes de amostras complexas, que ajusta as estimativas e seus erros-padrão considerando a estratificação, os conglomerados e os pesos amostrais) para amostras complexas, que incorpora o desenho amostral e os fatores de expansão da Pesquisa Nacional de Saúde 2019.

## Resultados

Foram analisados 22.705 indivíduos, dos quais 2.365 (11,8%) apresentaram depressão. Houve predominância do sexo feminino (12.523; 56,7%), maior concentração na faixa etária de 60 a 64 anos (6.802; 31,1%) e no ensino primário (14.966; 63,3%) (Tabela 1). A hipertensão arterial foi a condição crônica mais frequente (12.416; 56,4%), seguida por colesterol alto (5.864; 28,1%), diabetes (4.304; 20,8%), doença arterial coronariana (2.719; 13,1%) e acidente vascular cerebral (1.267; 5,6%). A prevalência de depressão foi maior no sexo feminino (1.794; 15,8%); diferença em relação ao masculino é estatisticamente significativa. Observou-se redução da prevalência com o avanço da idade (p-valor<0,001). Indivíduos com depressão apresentaram maior frequência de acidente vascular cerebral, doença arterial coronariana e colesterol alto em relação àqueles sem depressão (p-valor<0,001 para todas as comparações). 

**Tabela 1:** Características sociodemográficas e comorbidades dos idosos, segundo a presença de depressão. Brasil, 2019 (n=22.705)

Variáveis	Sem depressão	Depressão	Total^a^	p-valor
	n (%)	n (%)	n (%)	
Sexo				<0,001
Feminino	10.729 (84,2)	1.794 (15,8)	12.523 (56,7)	
Masculino	9.611 (93,4)	571 (6,6)	10.182 (43,3)	
Total	20.340 (88,2)	2.365 (11,8)	22.705 (100,0)	
Faixa etária				<0,001
60-64	6.032 (86,8)	770 (13,2)	6.802 (31,1)	
65-69	5.106 (87,2)	647 (12,8)	5.753 (25,3)	
70-74	3.794 (89,7)	418 (10,3)	4.212 (18,1)	
≥75	5.408 (89,8)	530 (10,2)	5.938 (25,5)	
Escolaridade				<0,001
Primário	13.540 (88,8)	1.426 (11,2)	14.966 (63,3)	
Secundário	4.698 (87,2)	633 (12,8)	5.331 (25,4)	
Superior	2.102 (87,0)	306 (13,0)	2.408 (11,3)	
Renda (em salários mínimos)				<0,001
0-2	2.184 (91,1)	208 (8,9)	2.392 (10,2)	
3-4	13.057 (88,7)	1.402 (11,3)	14.459 (63,3)	
≥5	5.099 (85,8)	755 (14,2)	5.854 (26,5)	
Co-residentes				<0,001
1-3	17.812 (89,4)	2.128 (10,6)	19.940 (87,8)	
4-6	2.338 (91,2)	229 (8,8)	2.567 (11,3)	
≥7	190 (96,3)	8 (3,7)	198 (0,9)	
Hipertensão arterial				<0,001
Não	9.276 (89,6)	855 (10,4)	10.131 (43,6)	
Sim	10.912 (87,3)	1.504 (12,7)	12.416 (56,4)	
Diabetes mellitus				<0,001
Não	15.896 (88,3)	1.805 (11,7)	17.701 (79,2)	
Sim	3.763 (86,5)	541 (13,5)	4.304 (20,8)	
Colesterol alto				<0,001
Não	14.693 (90,5)	1.338 (9,5)	16.031 (71,9)	
Sim	4.859 (81,3)	1.005 (18,7)	5.864 (28,1)	
Doença arterial coronariana				<0,001
Não	18.083 (89,3)	1.903 (10,7)	19.986 (86,9)	
Sim	2.257 (80,5)	462 (19,5)	2.719 (13,1)	
Acidente vascular cerebral				<0,001
Não	19.295 (88,7)	2.143 (11,3)	21.438 (94,4)	
Sim	1.045 (79,7)	222 (20,3)	1.267 (5,6)	

a As variações no total de observações devem-se a dados ignorados nas seguintes variáveis: Hipertensão arterial (n=158; 0,7%), Diabetes (n=700; 3,1%) e Colesterol alto (n=810; 3,6%).

No modelo de mensuração (Tabela 2), observou-se associação estatisticamente significativa entre a dimensão latente de comorbidades e seus indicadores observados. Em relação à hipertensão arterial (categoria de referência), foram estimados coeficientes mais elevados para diabetes (23,08; IC95% 15,83; 30,33), colesterol alto (15,80; IC95% 10,84; 20,76), doença arterial coronariana (37,78; IC95% 25,84; 49,72) e acidente vascular cerebral (160,83; IC95% 107,98; 213,68). Observa-se maior contribuição dessas condições para a construção da dimensão latente. No componente estrutural, verificou-se maior prevalência de depressão no sexo feminino (RP 2,82; IC95% 2,55; 3,12), enquanto a idade apresentou associação inversa com o desfecho em ambos os sexos (RP 0,98; IC95% 0,97; 0,99). A dimensão latente de comorbidades apresentou associação positiva com a depressão (RP 12,61; IC95% 11,59; 12,65). As razões de prevalência marginais preditivas por idade e sexo evidenciaram diferenças entre os sexos ao longo das faixas etárias analisadas (Figura 1).

**Tabela 2:** Coeficientes e intervalos de confiança de 95% (IC95%) da construção da dimensão latente de comorbidades. Brasil, 2019 (n=22.705)

Covariáveis	Coef^a^ (IC95%)	p-valor
Hipertensão arterial^c^	1,00	* *
Diabetes mellitus	23,08 (15,83; 30,33)	<0,001
Colesterol alto	15,80 (10,84; 20,76)	<0,001
Doença arterial coronariana	37,78 (25,84; 49,72)	<0,001
Acidente vascular cerebral	160,83 (107,98; 213,68)	<0,001
Variância	0,01 (0,01; 0,02)	
Covariáveis	RP^b^ (IC95%)	p-valor
Sexo		
Masculino^c^	1,00	
Feminino	2,82 (2,55; 3,12)	<0,001
Idade (anos)	0,98 (0,97; 0,99)	<0,001
Dimensão latente de comorbidades	12,61 (11,59; 12,65)	<0,001

a Coeficiente; ^b^Razão de prevalência; ^c^Variável de referência.

**Figura 1:**
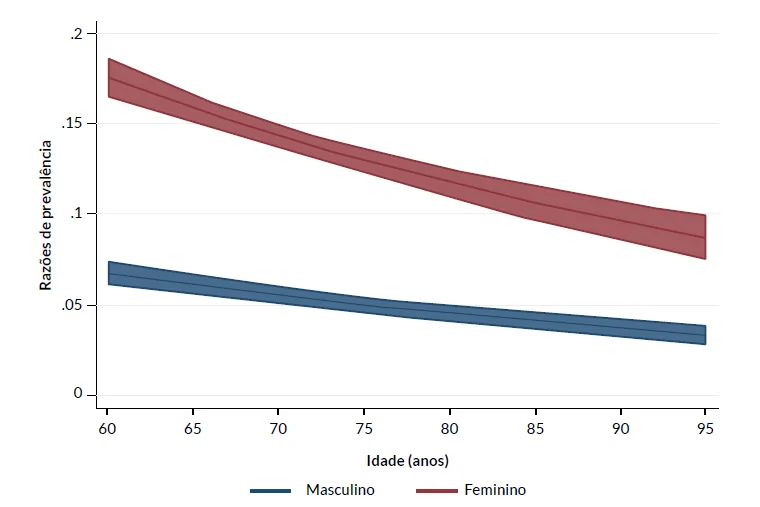
Razões de prevalência marginais para a ocorrência de depressão por sexo, segundo a idade (em anos). Brasil, 2019 (n=22.705)

Nos modelos estratificados por sexo (Tabela 3), o acidente vascular cerebral apresentou o maior coeficiente de carga fatorial no sexo masculino (29,29; IC95% 22,78; 35,81), seguido da doença arterial coronariana (10,64; IC95% 9,09; 12,18). Esse padrão é semelhante ao observado no sexo feminino, no qual o acidente vascular cerebral apresentou maior coeficiente (20,78; IC95% 19,35; 22,22), seguido da doença arterial coronariana (3,33; IC95% 3,07; 3,58). Observou-se associação inversa entre idade e depressão em ambos os sexos (masculino e feminino: RP 0,98; IC95% 0,97; 0,99), enquanto a renda apresentou associação positiva com o desfecho (masculino: RP 1,16; IC95% 1,10; 1,22; feminino: RP 1,11; IC95% 1,07; 1,14). Entre as mulheres, o número de corresidentes apresentou associação inversa com a depressão (RP 0,94; IC95% 0,91; 0,98). A dimensão latente de comorbidades apresentou associação positiva com a depressão em ambos os sexos, com média estimada de 12,20 (IC95% 11,56; 12,83) no sexo masculino e 7,61 (IC95% 5,51; 8,73) no feminino.

**Tabela 3:** Coeficientes, razões de prevalência (RP) e intervalos de confiança de 95% (IC95%) por sexo entre idosos. Brasil, 2019 (n=22.705)

Modelo de mensuração	Coef^a^ (IC95%)	p-valor
Sexo masculino		
Hipertensão arterial^c^	1,00	* *
Diabetes mellitus	7,26 (6,30; 8,12)	<0,001
Colesterol alto	6,80 (5,92; 7,68)	<0,001
Doença arterial coronariana	10,64 (9,09; 12,18)	<0,001
Acidente vascular cerebral	29,29 (22,78; 35,81)	<0,001
Variância	0,15 (0,12; 0,20)	
Sexo feminino		
Colesterol alto^c^	1,00	* *
Hipertensão arterial	-0,15 (-0,19; -0,10)	<0,001
Diabetes mellitus	1,86 (1,73; 1,99)	<0,001
Doença arterial coronariana	3,33 (3,07; 3,58)	<0,001
Acidente vascular cerebral	20,78 (19,35; 22,22)	<0,001
Variância	1,13 (1,00; 1,27)	
Modelo estrutural	RP^b^ (IC95%)	p-valor
Sexo masculino		
Idade (anos)	0,98 (0,97; 0,99)	<0,001
Renda (em salários mínimos)	1,16 (1,10; 1,22)	<0,001
Dimensão latente de comorbidades	12,20 (11,56; 12,83)	<0,001
Sexo feminino		
Idade (anos)	0,98 (0,97; 0,99)	<0,001
Nº de co-residentes	0,94 (0,91; 0,98)	<0,001
Renda (em salários mínimos)	1,11 (1,07; 1,14)	<0,001
Dimensão latente de comorbidades	7,61 (5,51; 8,73)	<0,001

a Coeficiente; ^b^Razão de prevalência; ^c^Variável de referência.

Entre as mulheres idosas, a dimensão latente de comorbidade apresentou a associação de maior magnitude com a depressão (β_4_ = 7,61), superior às associações observadas para as variáveis sociodemográficas: idade (β_1_ = 0,98), número de co-residentes (β_2_ = 0,94) e renda (β_3_ = 1,11). Entre os indicadores que compuseram a dimensão comorbidade, o acidente vascular cerebral apresentou a maior carga fatorial (γ_5_ = 20,75), seguido por doença arterial coronariana (γ_4_ = 3,33), diabetes mellitus (γ_3_ = 1,86) e colesterol alto (γ_1_ = 1,00); a hipertensão arterial foi o único indicador com carga negativa (γ_2_ = -0,15) (Figura 2).

**Figura 2:**
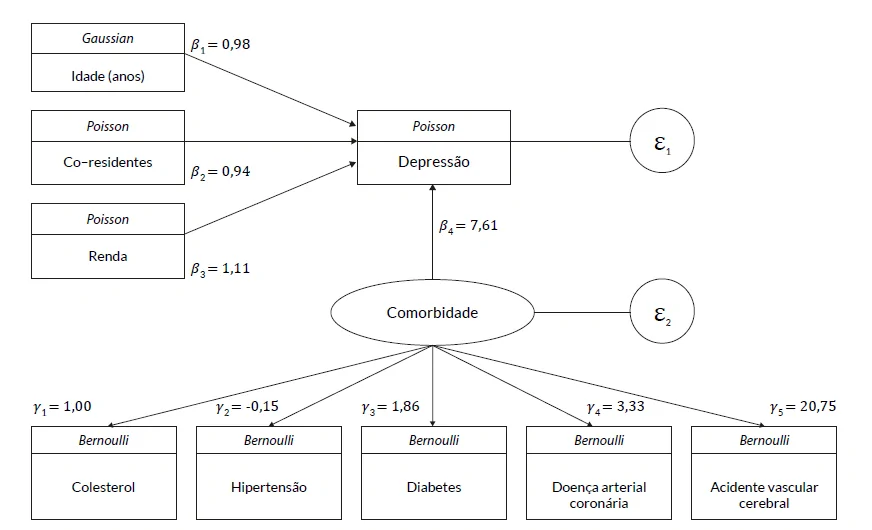
Modelo de dimensão latente de comorbidades em mulheres idosas. Brasil, 2019 (n=12.523)

## Discussão

Os resultados deste estudo, baseados em dados representativos da população idosa brasileira, indicaram prevalência de depressão de 11,8%, com maior ocorrência no sexo feminino. A dimensão latente de comorbidades, composta de múltiplas condições crônicas (hipertensão, diabetes, colesterol alto, doença arterial coronariana e acidente vascular cerebral), apresentou associação estatisticamente significativa com a depressão, mesmo após o ajuste por características sociodemográficas.

Esta análise apresenta limitações que devem ser consideradas na interpretação dos resultados. A utilização de dados secundários da PNS 2019 restringiu o controle sobre as variáveis coletadas e impediu a incorporação de informações não previstas no instrumento original [18]. Os dados foram obtidos por autorrelato de diagnóstico médico, método sujeito a viés de recordação e de informação, especialmente entre indivíduos com menor acesso aos serviços de saúde. O delineamento transversal não permitiu estabelecer precedência temporal entre as exposições e o desfecho, o que limitou as inferências de natureza causal. A ausência de variáveis clínicas detalhadas, como a gravidade das doenças, o tempo de diagnóstico e o uso de medicamentos, bem como de fatores comportamentais e psicossociais adicionais, pode ter restringido a avaliação de determinantes complementares. Adicionalmente, os dados foram coletados em 2019 e, considerando que a análise ocorre em 2026, há um intervalo de aproximadamente seis anos entre a coleta e o contexto atual. Eventos como a pandemia de covid-19 impactaram substancialmente a saúde mental dos idosos brasileiros nesse período [21], de modo que as estimativas podem não refletir integralmente o perfil epidemiológico vigente. Ainda assim, o tamanho amostral e a representatividade nacional da PNS conferem robustez às estimativas apresentadas.

A maior prevalência de depressão observada entre mulheres está em consonância com os achados de estudos nacionais e internacionais. Essa diferença tem sido atribuída a uma combinação de fatores biológicos, psicossociais e relacionados à trajetória de vida, incluindo desigualdades sociais persistentes e maior exposição a situações de vulnerabilidade ao longo do tempo [22,23,24]. 

Verificou-se associação inversa entre idade e depressão, com menor prevalência do desfecho nas faixas etárias mais avançadas, conforme identificado em estudos brasileiros baseados na Pesquisa Nacional de Saúde de 2019 [9,24]. Esse padrão é consistente com estimativas globais de carga de doença, referentes a 204 países e territórios no período de 1990 a 2019 [10]. Existem mecanismos adaptativos e maior resiliência psicológica entre indivíduos de idades mais elevadas [15,24]. Entretanto, essa relação pode variar conforme o contexto sociocultural e o método de diagnóstico empregado [10,22], o que reforça a importância de investigações representativas no cenário nacional [9,15,23].

A dimensão latente de comorbidades apresentou forte associação com a depressão em ambos os sexos. Esse achado apontam ao fato de que essa multimorbidade constitui um fator relevante no comprometimento da saúde mental em idosos [25,26]. Uma revisão abrangendo 72 estudos e 114 metanálises demonstrou que indivíduos com duas ou mais doenças crônicas apresentam maior risco de prejuízos nos indicadores de bem-estar mental [27]. No presente estudo, condições como doença arterial coronariana, acidente vascular cerebral, hipertensão, colesterol alto e diabetes contribuíram significativamente para a composição da dimensão latente, destacando o papel central do acidente vascular cerebral na estrutura do construto. Tais condições têm sido consistentemente associadas à maior prevalência de sintomas depressivos [9,26].

A renda e o suporte social, mensurados pelo número de corresidentes, também se associaram à depressão. Níveis mais elevados de renda estiveram associados a maior prevalência de depressão [23,26]. Esse achado pode refletir especificidades do contexto brasileiro, como maior acesso ao diagnóstico e maior reconhecimento de sintomas depressivos em grupos socioeconômicos mais favorecidos. O efeito protetor observado no maior número de corresidentes sugere a relevância do suporte familiar para a saúde mental da população idosa [28].

Nesta análise, aplicar equações estruturais generalizadas possibilitou uma análise integrada e aprofundada dos fatores associados à depressão, favorecendo a identificação mais precisa de seus determinantes entre pessoas idosas. Estratégias analíticas hierarquizadas têm se mostrado úteis na investigação desses fatores, especialmente em contextos de envelhecimento populacional, em que a prevalência da depressão tende a aumentar e seus determinantes tornam-se mais complexos [29]. A abordagem adotada permitiu estimar o impacto da dimensão latente de comorbidades sobre a ocorrência do desfecho, testar empiricamente a adequação do modelo teórico proposto e caracterizar o efeito conjunto de múltiplas comorbidades, com ajuste simultâneo para variáveis sociodemográficas [30].

Do ponto de vista da organização do cuidado no Sistema Único de Saúde, os achados indicam a necessidade de integrar o manejo de condições crônicas às ações de saúde mental voltadas à população idosa. A associação entre a dimensão latente de comorbidades e a depressão reforça a importância do rastreamento sistemático de sintomas depressivos na atenção primária à saúde, especialmente entre idosos com doenças crônicas, bem como da articulação com a rede de atenção psicossocial. Em um contexto de rápido envelhecimento populacional, estratégias integradas podem contribuir para a redução do sofrimento psíquico e para a melhoria da qualidade de vida.

A depressão em idosos brasileiros mostrou-se associada à presença simultânea de múltiplas condições crônicas, ao sexo feminino, à idade e a fatores socioeconômicos. A utilização de uma dimensão latente de comorbidades ampliou a compreensão das interações entre doenças crônicas e a saúde mental, oferecendo subsídios para o planejamento de políticas públicas e de práticas assistenciais direcionadas à população idosa.

## MATERIAL SUPLEMENTAR

MATERIAL SUPLEMENTAR

## Data Availability

Os dados utilizados neste estudo são públicos e estão disponíveis no site do Instituto Brasileiro de Geografia e Estatística, por meio da Pesquisa Nacional de Saúde 2019: https://www.ibge.gov.br/estatisticas/sociais/saude/9160-pesquisa-nacional-de-saude.html?=&t=microdados. Os dados tratados e o código da análise podem ser disponibilizados pelos autores mediante solicitação.
