# Supplementary material for: Dimensão latente de comorbidades e depressão em idosos: estudo transversal com dados da Pesquisa Nacional de Saúde, Brasil, 2019
Source: Epidemiol Serv Saude. 2026 Oct 12;35:e20250722. [Article in Portuguese] doi: 10.1590/S2237-96222026v35e20250722.pt (PMC13671081; doi:10.1590/S2237-96222026v35e20250722.pt)
Supplement: Supplementary file 1 [file 2237-9622-ress-35-e20250722-md1-en.pdf]

Supplementary Table 1. Description of the modules, variables, questions and categories used in the study. National Health Survey (*Pesquisa Nacional de Saúde*, PNS). Brazil, 2019

Module	Variables	PNS 2029 questionnaire questions PNS 2019	Categories used in the study
C – General characteristics of household dwellers	Sex	C006. Sex	Male; Female
	Age group	C008. Age in years	60-64; 65-69; 70-74; ≥75
	Co-residents	C001. Number of people in the household	1-3; 4-6; ≥7
Variables derived from Modules D and F	Schooling	VDD004A. Highest level of education achieved (people aged 5 years or over) standardized to Elementary Education – 9 YEAR SYSTEM	Primary (incomplete, complete elementary or equivalent); Secondary (incomplete, complete high school or equivalent); Higher Education (incomplete, complete or equivalent)
	Income (minimum wages)	VDF004. Per capita household income range (excluding the income of persons whose status within the household was boarder, domestic worker or relative of the domestic worker)	0-2; 3-4; ≥5
Q – Doenças crônicas	Hypertension	Q00201. Has a doctor ever diagnosed you as having hypertension (high blood pressure)?	No; Yes
	Diabetes mellitus	Q03001. Has a doctor ever diagnosed you as having diabetes?	No; Yes
	High cholesterol	Q060. Has a doctor ever told you that you have high cholesterol?	No; Yes
	Coronary artery disease	Q063. Has a doctor ever diagnosed you as having a heart disease, such as infarction (blockage), angina, heart failure or other heart disease?	No; Yes
	Stroke	Q068. Has a doctor ever diagnosed you as having had a stroke?	No; Yes
	Depression (outcome)	Q092. Has a doctor or a health professional (such as a psychiatrist or psychologist) ever diagnosed you as having depression?	No; Yes

Note: All health variables were obtained via self-reporting in Module Q, based on a prior diagnosis by a physician or mental health professional communicated to the respondent. Depression was defined as the presence of self-reported diagnosis and served as the primary outcome in the GSEM model (modeled using Poisson regression to estimate prevalence ratios). Please consult the full questionnaire at https://www.pns.icict.fiocruz.br/ for further details.

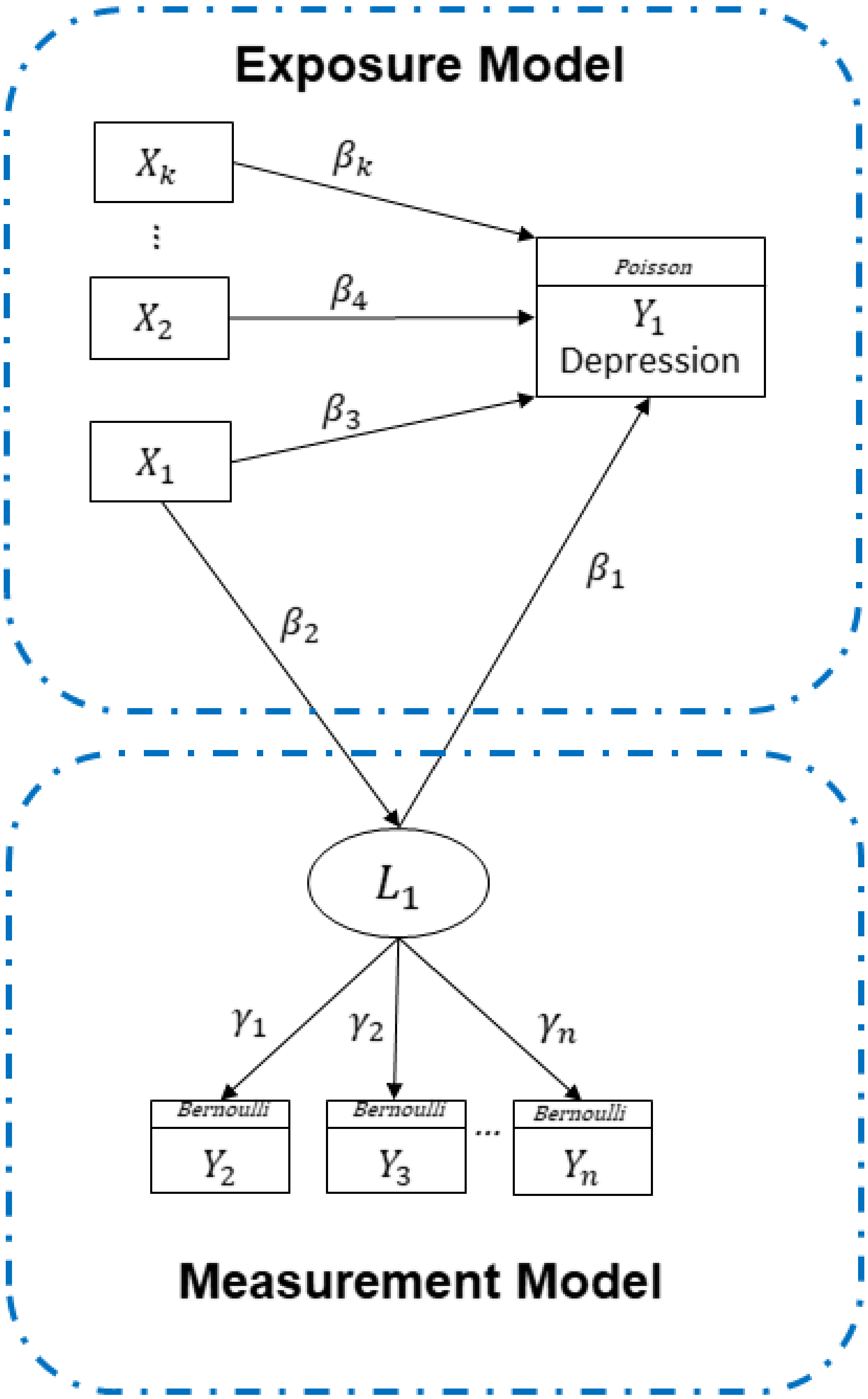

Supplementary Figure 1. Conceptual model of chronic diseases associated with occurrence of depression in older adults. Brazil, 2019
