# Supplementary material for: Dimensão latente de comorbidades e depressão em idosos: estudo transversal com dados da Pesquisa Nacional de Saúde, Brasil, 2019
Source: Epidemiol Serv Saude. 2026 Oct 12;35:e20250722. [Article in Portuguese] doi: 10.1590/S2237-96222026v35e20250722.pt (PMC13671081; doi:10.1590/S2237-96222026v35e20250722.pt)
Supplement: Supplementary file 2 [file 2237-9622-ress-35-e20250722-md1.pdf]

Tabela Suplementar 1. Descrição dos módulos, variáveis, perguntas e categorias utilizadas no estudo. Pesquisa Nacional de Saúde (PNS). Brasil, 2019

Módulo	Variáveis	Perguntas do questionário da PNS 2019	Categorias utilizadas no estudo
C – Características gerais dos moradores	Sexo	C006. Sexo	Masculino; Feminino
	Faixa etária	C008. Idade em anos	60-64; 65-69; 70-74; ≥75
	Co-residentes	C001. Número de pessoas no domicílio	1-3; 4-6; ≥7
Variáveis derivadas dos Módulos D e F	Escolaridade	VDD004A. Nível de instrução mais elevado alcançado (pessoas de 5 anos ou mais de idade) padronizado para o Ensino Fundamental - SISTEMA DE 9 ANOS	Primário (fundamental incompleto, completo ou equivalente); Secundário (médio incompleto, completo ou equivalente); Superior (incompleto, completo ou equivalente)
	Renda (salários mínimos)	VDF004. Faixa de rendimento domiciliar per capita (exclusive o rendimento das pessoas cuja condição na unidade domiciliar era pensionista, empregado doméstico ou parente do empregado doméstico)	0-2; 3-4; ≥5
Q – Doenças crônicas	Hipertensão arterial	Q00201. Algum médico já lhe deu o diagnóstico de hipertensão arterial (pressão alta)?	Não; Sim
	Diabetes mellitus	Q03001. Algum médico já lhe deu o diagnóstico de diabetes?	Não; Sim
	Colesterol alto	Q060. Algum médico já lhe disse que você tem colesterol alto?	Não; Sim
	Doença arterial coronariana	Q063. Algum médico já lhe deu o diagnóstico de uma doença do coração, tal como infarto, angina, insuficiência cardíaca ou outra?	Não; Sim
	Acidente vascular cerebral	Q068. Algum médico já lhe deu o diagnóstico de AVC (Acidente Vascular Cerebral) ou derrame?	Não; Sim
	Depressão (desfecho)	Q092. Algum médico ou profissional de saúde mental (como psiquiatra ou psicólogo) já lhe deu o diagnóstico de depressão?	Não; Sim

Nota: Todas as variáveis de saúde foram obtidas por autorrelato no Módulo Q, com base em diagnóstico médico ou de profissional de saúde mental prévio comunicado ao respondente. A depressão foi definida como presença de diagnóstico autorreferido, servindo como desfecho principal no modelo GSEM (modelado como Poisson para estimar razões de prevalência). Consulte o questionário completo em https://www.pns.icict.fiocruz.br/ para obter detalhes mais precisos.

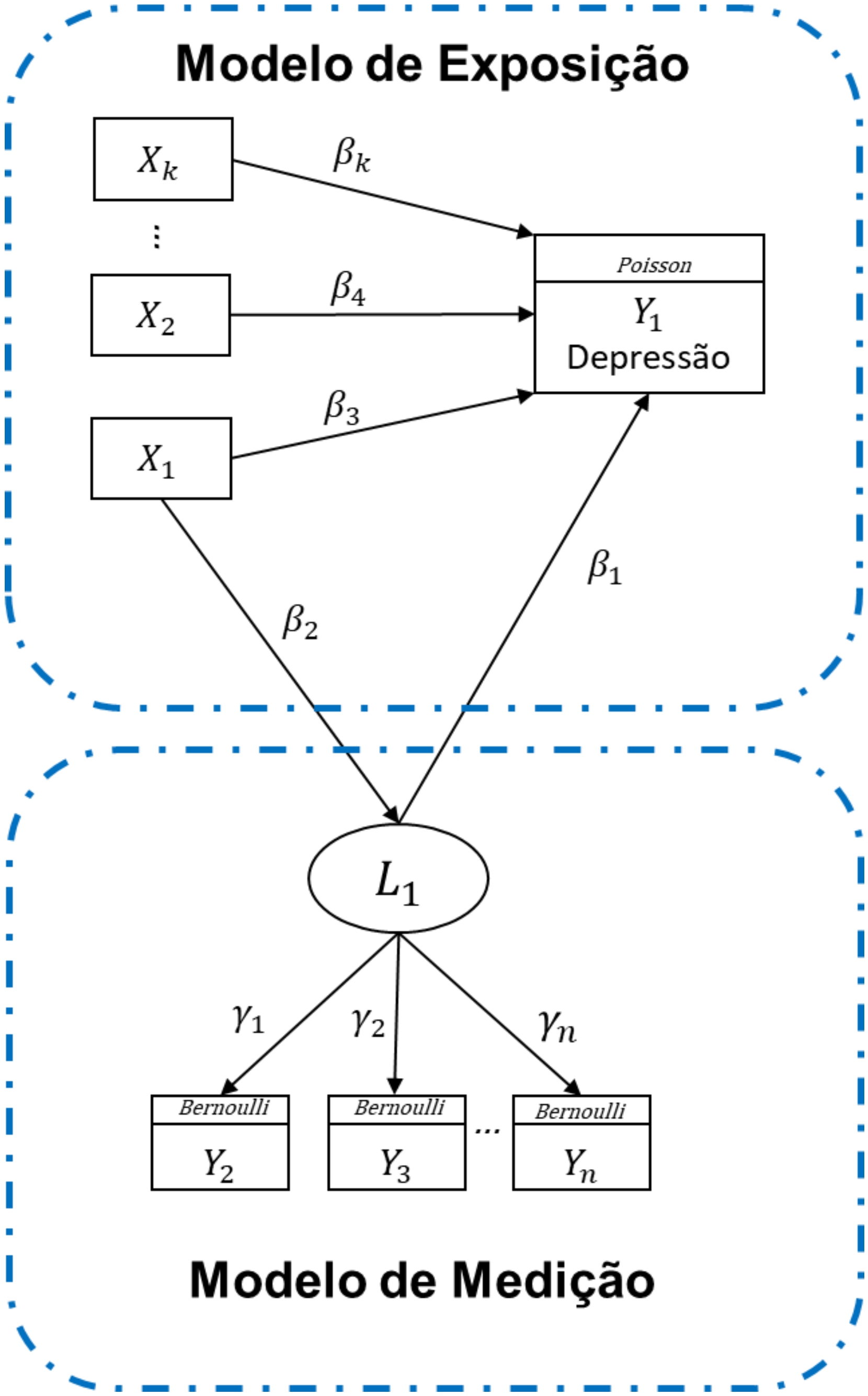

Figura Suplementar 1. Modelo conceitual de doenças crônicas associadas à ocorrência de depressão em idosos. Brasil, 2019
