# Supplementary material for: Dimensão latente de comorbidades e depressão em idosos: estudo transversal com dados da Pesquisa Nacional de Saúde, Brasil, 2019
Source: Epidemiol Serv Saude. 2026 Oct 12;35:e20250722. [Article in Portuguese] doi: 10.1590/S2237-96222026v35e20250722.pt (PMC13671081; doi:10.1590/S2237-96222026v35e20250722.pt)
Supplement: Translation

# Translation: Latent dimension of comorbidities and depression in older adults: a cross-sectional study using National Health Survey data, Brazil, 2019

**DOI:** 10.1590/S2237-96222026v35e20250722.en

**Authors:** Fabian Leonardo Muñoz, Renato Ferreira da Cruz, Liciana Vaz de Arruda Silveira, Maria del Pilar Diaz

**Affiliations:** 1 Universidad Nacional de Córdoba, Facultad de Ciencias Médicas, Córdoba, Argentina; 2 Universidade Federal de Mato Grosso, Barra do Garças, MT, Brazil; 3 Universidade Estadual Paulista, Instituto de Biociências, Botucatu, SP, Brazil

**Keywords:** Depression, Comorbidity, Aged, Socioeconomic Factors, Cross-Sectional Studies

## Abstract

**Objective:**

To build a latent dimension of comorbidities and assess its association with depression among older Brazilian adults.

**Methods:**

This was a cross-sectional study using Brazilian National Health Survey 2019 data, including individuals aged 60 years or older. The latent dimension of comorbidities was built based on five self-reported conditions: hypertension, diabetes mellitus, high cholesterol, coronary artery disease and stroke. Generalized Structural Equation Modeling (GSEM) was employed. Prevalence ratios (PR) and 95% confidence intervals (95%CI) were estimated using Poisson regression with robust variance.

**Results:**

A total of 22,705 older adults were analyzed, whereby prevalence of depression was 11.8%. Hypertension was the most frequent condition (56.4%). The latent dimension of comorbidities showed strong positive association with depression (PR 12.61; 95%CI 11.59; 12.65). Outcome prevalence was higher among women (PR 2.82; 95%CI 2.55; 3.12) and those with higher income (PR 1.11-1.16), whereas advancing age was inversely associated with depression (PR 0.98; 95%CI 0.97; 0.99).

**Conclusion:**

Depression among older Brazilian adults was significantly associated with comorbidity burden, female sex, age and income. Latent dimension modeling contributed to an integrated understanding of chronic conditions, revealing that the accumulation of these pathologies has a substantial impact on the mental health of this population, reinforcing the need for multiprofessional and integrated approaches to elderly care within the Brazilian Unified Health System.

## Introduction

Depression is a major global public health problem; it contributes to loss of functionality and increased disease burden, particularly among older adults. It is estimated that approximately 332 million people worldwide live with this condition, making it one of the most common mental disorders [1,2]. In Brazil, it is one of the most common and recurrent mental disorders, negatively impacting quality of life and increasing mortality risk [3,4].

In old age, depression stands out as one of the most common mental disorders, characterized by persistently low mood, apathy, reduced energy, changes in sleep and body weight and cognitive impairments such as difficulty concentrating and psychomotor slowing. There may also be a decrease in libido, feelings of guilt, negative thoughts, and, in severe cases, suicidal ideation [5]. It is a condition that generally follows a prolonged course and is influenced by multiple determinants, including biological, psychological, social and environmental factors [6]. Although its etiology is not fully understood, there is consensus regarding the contribution of psychosocial and biological factors, as well as its association with other psychiatric diagnoses, such as anxiety and personality disorders [7]. In the elderly, the clinical picture tends to be more complex, often resulting from interaction between vulnerabilities accumulated over a lifetime and challenges specific to this age group [8].

Prevalence of depression is higher among women [3,9] and is associated with advancing age, lower levels of education, low income, loneliness or living alone, and presence of chronic diseases or poor health status, among other factors [10].

In Brazil, population aging has been occurring at an accelerated pace. The number of people aged 60 or older rose from 20.6 million in 2010 to 32.1 million in 2022, representing a 56% increase in just over a decade [11]. Advances in medicine have reduced mortality and increased life expectancy but have also raised the burden of chronic non-communicable diseases, such as hypertension, diabetes and cardiovascular diseases, which coexist and negatively affect mental health [12]. This new demographic landscape is significant for defining rights and benefits for the elderly. It is essential to acknowledge the specific needs and challenges associated with aging, such as multimorbidity, functional limitations, low income, abandonment and violence [13,14].

In Brazil, 13% prevalence of clinically relevant depressive symptoms among older adults was recorded in 2019 [9]. These data reveal significant variations based on sex, age and socioeconomic conditions, highlighting a higher burden of the condition among women and individuals with lower levels of education [9,15]. Conversely, in 2022, lower prevalence of depression was observed among older adults residing in rural areas and in the Northeast and Northern regions of Brazil [16]. Such geographic differences may reflect sociocultural specificities, family structures, local support networks and distinct perceptions of health and illness, all of which influence the expression and recognition of depressive symptoms [17].

Coexistence of multiple chronic diseases in older adults limits analyses based on isolated variables, given that these conditions tend to occur in an interrelated manner. In order to address this complexity, Generalized Structural Equation Modeling (GSEM) has been employed, this being an approach that integrates measurement and structural components into a single analytical framework, enabling a latent dimension to be built that synthesizes the overall burden of comorbidities. The objective of this study was to build a latent dimension of comorbidities and assess its association with depression among older Brazilian adults.

## Methods

### Design

This is a population-based, cross-sectional study with an analytical design, conducted using secondary data from the 2019 National Health Survey (*Pesquisa Nacional de Saúde*, PNS). The study was prepared in accordance with the guidelines of the Strengthening the Reporting of Observational Studies in Epidemiology (STROBE) statement.

### Setting

PNS is a nationwide household survey conducted by the Brazilian Institute of Geography and Statistics (IBGE) in partnership with the Ministry of Health. This study used data collected in 2019 [18]. The survey is part of the IBGE’s Integrated System of Household Surveys and aims to produce robust estimates regarding multiple dimensions of the Brazilian population’s health status. Its thematic scope encompasses self-reported health conditions, chronic morbidities, lifestyle-related behaviors, health service use, and health prevention and promotion activities. It offers national geographic coverage, with representativeness across major regions, Federative Units, metropolitan areas and state capital cities [18].

### Participants

The target population of this study consisted of individuals aged 60 years or older residing in private households throughout Brazil. Individuals with complete information on the outcome variable (depression) and exposure variables (the chronic conditions investigated) were included.

The PNS 2019 sample was selected using a three-stage probability cluster sampling design. In the first stage, census tracts or sets of tracts (primary sampling units) were selected; in the second, permanent private households within those tracts; and in the third, one adult resident was randomly selected from each household to answer the individual questionnaire.

For this study, only individuals aged 60 years or older who participated in the individual module of PNS were included. The interviews were conducted by previously trained personnel using standardized questionnaires [18].

### Data sources

The data were extracted from the 2019 PNS public database, available at https://www.pns.icict.fiocruz.br/. Sociodemographic variables were obtained from module C (General characteristics of residents), module D (Education characteristics), and module F (Household income). Data on chronic conditions and depression were obtained from module Q (Chronic diseases), based on self-reported prior medical diagnoses. All information was collected via self-reporting during in-person interviews.

### Variables

The outcome variable was depression, defined by a self-reported diagnosis previously given by a physician or mental health professional (psychiatrist or psychologist), obtained via the question: “Has a physician or mental health professional ever diagnosed you as having depression?” The outcome was considered present when the response was “yes.”

The primary exposure variable was the latent dimension of comorbidities, operationalized via a Generalized Structural Equation Model (GSEM) and built based on five self-reported chronic conditions: hypertension (yes; no), diabetes (yes; no), high cholesterol (yes; no), coronary artery disease, including acute myocardial infarction or angina (yes; no), and stroke (yes; no). The latent variable was specified taking hypertension as the reference indicator (loading=1.00), allowing for the free estimation of coefficients for the other conditions in order to identify the differential contribution of each morbidity. Explanatory variables included sociodemographic characteristics: sex (male; female), age group (60-64; 65-69; 70-74; ≥75 years), schooling (primary; secondary; higher education), household income (0-2; 3-4; ≥5 ​​minimum wages), and number of co-residents (1-3; 4-6; ≥7 household members). Variable categorization was based on approaches widely used in epidemiological studies, aiming to capture different risk levels and ensure the robustness of the estimates (Supplementary Table 1).

### Bias

Information bias inherent to self-reporting is acknowledged, as it may underestimate prevalence rates among individuals with limited access to health services. The cross-sectional design precludes the establishment of temporal precedence, posing a risk of reverse causality. Survivorship bias and residual confounding due to unmeasured variables are also plausible. In order to mitigate selection bias, all analyses incorporated the expansion factors and the complex sample design of the PNS.

### Study size

The sample size was not determined by a specific calculation for this study, as the analysis included all eligible participants from the 2019 PNS who were aged 60 years or older and had complete data for all variables of interest. A total of 22,705 individuals were included.

### Statistical methods

Initially, a descriptive analysis was performed, including absolute and relative frequency distributions, point estimates and 95% confidence intervals (95%CI). Differences in proportions between individuals with and without depression were assessed using the chi-square test, taking a 5% significance level.

The primary exposure variable was operationalized as the latent dimension of comorbidities, structured within a GSEM framework comprising two simultaneous components: the measurement component, to build the latent dimension, and the structural component, to estimate association with depression while adjusting for sociodemographic covariates. Effects were estimated using maximum likelihood. The latent dimension of comorbidities is represented by an ellipse, and the observed variables by rectangles, with arrows indicating the linear effects (γ_n_) between them.

Poisson regression with robust variance [19] was applied in order to obtain prevalence ratios (PR) and their respective 95%CIs. This method is suitable for cross-sectional studies with binary outcomes. In addition to the general model, sex-stratified analyses were conducted, and predictive marginal prevalence ratios by age were calculated. The final model selection was based on Akaike and Bayesian information criteria, prioritizing goodness-of-fit and theoretical coherence [20]. Cases with missing information were excluded from specific analyses, without data imputation.

The analyses were performed using Data Analysis and Statistical Software (Stata), version 17.0, utilizing the survey module for complex samples (a set of commands within the statistical software designed to analyze data from complex samples, adjusting estimates and their standard errors by accounting for stratification, clustering and sampling weights) which incorporates the sample design and expansion factors of the 2019 National Health Survey.

## Results

A total of 22,705 individuals were analyzed, of whom 2,365 (11.8%) presented depression. There was a predominance of females (12,523; 56.7%), with the highest concentration in the 60-64 age group (6,802; 31.1%) and among those with primary education (14,966; 63.3%) (Table 1). Hypertension was the most frequent chronic condition (12,416; 56.4%), followed by high cholesterol (5,864; 28.1%), diabetes (4,304; 20.8%), coronary artery disease (2,719; 13.1%) and stroke (1,267; 5.6%). Prevalence of depression was higher among females (1,794; 15.8%); the difference compared to males was statistically significant. A decrease in prevalence was observed as age advanced (p-value<0.001). Individuals with depression showed a higher frequency of stroke, coronary artery disease and high cholesterol compared to those without depression (p-value<0.001 for all comparisons).

**Table 1:** Older adult sociodemographic characteristics and comorbidities, according to presence of depression. Brazil, 2019 (n=22,705)

Variables	Without depression	Depression	Total^a^	p-value
	n (%)	n (%)	n (%)	
Sex				<0.001
Female	10,729 (84.2)	1,794 (15.8)	12,523 (56.7)	
Male	9,611 (93.4)	571 (6.6)	10,182 (43.3)	
Total	20,340 (88.2)	2,365 (11.8)	22,705 (100.0)	
Age group				<0.001
60-64	6,032 (86.8)	770 (13.2)	6,802 (31.1)	
65-69	5,106 (87.2)	647 (12.8)	5,753 (25.3)	
70-74	3,794 (89.7)	418 (10.3)	4,212 (18.1)	
≥75	5,408 (89.8)	530 (10.2)	5,938 (25.5)	
Schooling				<0.001
Primary	13,540 (88.8)	1,426 (11.2)	14,966 (63.3)	
Secondary	4,698 (87.2)	633 (12.8)	5,331 (25.4)	
Higher education	2,102 (87.0)	306 (13.0)	2,408 (11.3)	
Income (in minimum wages)				<0.001
0-2	2,184 (91.1)	208 (8.9)	2,392 (10.2)	
3-4	13,057 (88.7)	1,402 (11.3)	14,459 (63.3)	
≥5	5,099 (85.8)	755 (14.2)	5,854 (26.5)	
Co-residents				<0.001
1-3	17,812 (89.4)	2,128 (10.6)	19,940 (87.8)	
4-6	2,338 (91.2)	229 (8.8)	2,567 (11.3)	
≥7	190 (96.3)	8 (3.7)	198 (0.9)	
Hypertension				<0.001
No	9,276 (89.6)	855 (10.4)	10,131 (43.6)	
Yes	10,912 (87.3)	1,504 (12.7)	12,416 (56.4)	
Diabetes mellitus				<0.001
No	15,896 (88.3)	1,805 (11.7)	17,701 (79.2)	
Yes	3,763 (86.5)	541 (13.5)	4,304 (20.8)	
High cholesterol				<0.001
No	14,693 (90.5)	1,338 (9.5)	16,031 (71.9)	
Yes	4,859 (81.3)	1,005 (18.7)	5,864 (28.1)	
Coronary artery disease				<0.001
No	18,083 (89.3)	1,903 (10.7)	19,986 (86.9)	
Yes	2,257 (80.5)	462 (19.5)	2,719 (13.1)	
Stroke				<0.001
No	19,295 (88.7)	2,143 (11.3)	21,438 (94.4)	
Yes	1,045 (79.7)	222 (20.3)	1,267 (5.6)	

a The variations in total observations are due to missing data in the following variables: Hypertension (n=158; 0.7%) Diabetes (n=700; 3.1%) and High cholesterol (n=810; 3.6%).

In the measurement model (Table 2), statistically significant association was observed between the latent dimension of comorbidities and its observed indicators. Compared to hypertension (the reference category), higher coefficients were estimated for diabetes (23.08; 95%CI 15.83; 30.33), high cholesterol (15.80; 95%CI 10.84; 20.76), coronary artery disease (37.78; 95%CI 25.84; 49.72) and stroke (160.83; 95%CI 107.98; 213.68). These conditions were found to make a greater contribution to the building of the latent dimension. In the structural component, higher prevalence of depression was observed among females (PR 2.82; 95%CI 2.55; 3.12), whereas age showed inverse association with the outcome in both sexes (PR 0.98; 95%CI 0.97; 0.99). The latent dimension of comorbidities showed positive association with depression (PR 12.61; 95%CI 11.59; 12.65). Predictive marginal prevalence ratios by age and sex revealed differences between the sexes across the age groups analyzed (Figure 1).

**Table 2:** Coefficients and 95% confidence intervals (95%CI) used to build the latent dimension of comorbidities. Brazil, 2019 (n=22,705)

Covariables	Coef^a^ (95%CI)	p-value
Hypertension^c^	1.00	* *
Diabetes mellitus	23.08 (15.83; 30.33)	<0.001
High cholesterol	15.80 (10.84; 20.76)	<0.001
Coronary artery disease	37.78 (25.84; 49.72)	<0.001
Stroke	160.83 (107.98; 213.68)	<0.001
Variance	0.01 (0.01; 0.02)	
Covariables	RP^b^ (95%CI)	p-value
Sex		
Male^c^	1.00	
Female	2.82 (2.55; 3.12)	<0.001
Age (years)	0.98 (0.97; 0.99)	<0.001
Latent dimension of comorbidities	12.61 (11.59; 12.65)	<0.001

a Coefficient; ^b^Prevalence ratio; ^c^Reference variable.

**Figure 1:** Marginal prevalence ratios for occurrence of depression, by sex, according to age (in years). Brazil, 2019 (n=22,705)

In the sex-stratified models (Table 3), stroke showed the highest factor loading coefficient among males (29.29; 95%CI 22.78; 35.81), followed by coronary artery disease (10.64; 95CI 9.09; 12.18). This pattern is similar to that observed among females, where stroke showed the highest coefficient (20.78; 95%CI 19.35; 22.22), followed by coronary artery disease (3.33; 95% CI 3.07; 3.58). Inverse association was observed between age and depression in both sexes (males and females: PR 0.98; 95%CI 0.97; 0.99), whereas income showed positive association with the outcome (males: PR 1.16; 95%CI 1.10; 1.22; females: PR 1.11; 95%CI 1.07; 1.14). Among women, the number of co-residents showed inverse association with depression (PR 0.94; 95% CI 0.91; 0.98). The latent dimension of comorbidities showed positive association with depression in both sexes, with an estimated mean of 12.20 (95%CI 11.56; 12.83) for males and 7.61 (95%CI 5.51; 8.73) for females.

**Table 3:** Coefficients, prevalence ratios (PR) and 95% confidence intervals (95%CI) according to sex, among elderly adults. Brazil, 2019 (n=22,705)

Measurement model	Coef^a^ (95%CI)	p-value
Males		
Hypertension^c^	1.00	* *
Diabetes mellitus	7.26 (6.30; 8.12)	<0.001
High cholesterol	6.80 (5.92; 7.68)	<0.001
Coronary artery disease	10.64 (9.09; 12.18)	<0.001
Stroke	29.29 (22.78; 35.81)	<0.001
Variance	0.15 (0.12; 0.20)	
Females		
High cholesterol^c^	1.00	* *
Hypertension	-0.15 (-0.19; -0.10)	<0.001
Diabetes mellitus	1.86 (1.73; 1.99)	<0.001
Coronary artery disease	3.33 (3.07; 3.58)	<0.001
Stroke	20.78 (19.35; 22.22)	<0.001
Variance	1.13 (1.00; 1.27)	
Structural model	PR^b^ (95%CI)	p-value
Males		
Age (years)	0.98 (0.97; 0.99)	<0.001
Income (in minimum wages)	1.16 (1.10; 1.22)	<0.001
Latent dimension of comorbidities	12.20 (11.56; 12.83)	<0.001
Females		
Age (years)	0.98 (0.97; 0.99)	<0.001
Number of co-residents	0.94 (0.91; 0.98)	<0.001
Income (in minimum wages)	1.11 (1.07; 1.14)	<0.001
Latent dimension of comorbidities	7.61 (5.51; 8.73)	<0.001

a Coefficient; ^b^Prevalence ratio; ^c^Reference variable.

Among older women, the latent comorbidity dimension showed the strongest association with depression (β₄ = 7.61), exceeding the associations observed for sociodemographic variables: age (β₁ = 0.98), number of co-residents (β₂ = 0.94), and income (β₃ = 1.11). Among the indicators comprising the comorbidity dimension, stroke showed the highest factor loading (γ₅ = 20.75), followed by coronary artery disease (γ₄ = 3.33), diabetes mellitus (γ₃ = 1.86), and high cholesterol (γ₁ = 1.00); hypertension was the only indicator with a negative loading (γ₂ = -0.15) (Figure 2).

**Figure 2:** Model of latent dimension of comorbidities in older adult women. Brazil, 2019 (n=12,523)

## Discussion

The results of this study, based on data representative of the Brazilian elderly population, indicated a depression prevalence of 11.8%, with higher occurrence among females. The latent dimension of comorbidities, comprising multiple chronic conditions (hypertension, diabetes, high cholesterol, coronary artery disease and stroke), showed statistically significant association with depression, even after adjustment for sociodemographic characteristics.

This analysis has limitations that should be considered when interpreting the results. Use of secondary data from the 2019 PNS restricted control over the variables collected and precluded inclusion of information not covered by the original instrument [18]. Data were obtained via self-reported medical diagnoses, a method susceptible to recall and information bias, particularly among individuals with limited access to healthcare services. The cross-sectional design did not allow for the establishment of temporal precedence between exposures and the outcome, thereby limiting causal inferences. The absence of detailed clinical variables, such as disease severity, time since diagnosis and medication use, as well as additional behavioral and psychosocial factors, may have restricted assessment of complementary determinants. Furthermore, the data were collected in 2019; given that this analysis is being conducted in 2026, there is a gap of approximately six years between data collection and the current context. Events such as the COVID-19 pandemic substantially impacted the mental health of older Brazilians during this period [21], meaning the estimates may not fully reflect the current epidemiological profile. Nevertheless, the sample size and national representativeness of the PNS lend robustness to the estimates presented.

The higher prevalence of depression observed among women is consistent with findings from national and international studies. This difference has been attributed to a combination of biological, psychosocial and life-course factors, including persistent social inequalities and greater exposure to situations of vulnerability over time [22,23,24]. 

Inverse association was observed between age and depression, with lower prevalence of the outcome in older age groups, as identified in Brazilian studies based on the 2019 National Health Survey [9,24]. This pattern is consistent with global disease burden estimates covering 204 countries and territories between 1990 and 2019 [10]. Adaptive mechanisms and greater psychological resilience exist among older individuals [15,24]. However, this relationship may vary depending on the sociocultural context and the diagnostic method employed [10,22], underscoring the importance of representative studies at the national level [9,15,23].

The latent dimension of comorbidities showed strong association with depression in both sexes. This finding indicates that multimorbidity is a significant factor in the impairment of mental health among older adults [25,26]. A review covering 72 studies and 114 meta-analyses demonstrated that individuals with two or more chronic diseases are at higher risk of adverse effects on mental well-being indicators [27]. In the present study, conditions such as coronary artery disease, stroke, hypertension, high cholesterol and diabetes contributed significantly to the composition of the latent dimension, highlighting the central role of stroke in the construct’s structure. These conditions have been consistently associated with higher prevalence of depressive symptoms [9,26].

Income and social support, measured by the number of co-residents, were also associated with depression. Higher income levels have been associated with higher prevalence of depression [23,26]. This finding may reflect specific aspects of the Brazilian context, such as greater access to diagnosis and increased recognition of depressive symptoms among more socioeconomically advantaged groups. The protective effect observed with a higher number of co-residents suggests the importance of family support for the mental health of the elderly population [28].

In this analysis, applying generalized structural equations enabled an integrated and in-depth examination of factors associated with depression, facilitating a more precise identification of its determinants among older adults. Hierarchical analytical strategies have proven useful in investigating these factors, particularly in the context of population aging, where the prevalence of depression tends to rise and its determinants become increasingly complex [29]. The approach adopted made it possible to estimate the impact of the latent dimension of comorbidities on the outcome, empirically test the fit of the proposed theoretical model, and characterize the combined effect of multiple comorbidities while simultaneously adjusting for sociodemographic variables [30].

From the perspective of care organization within the Brazilian Unified Health System, the findings indicate a need to integrate management of chronic conditions with mental health interventions aimed at the elderly population. The association between the latent dimension of comorbidities and depression underscores the importance of systematically screening for depressive symptoms in primary health care, particularly among older adults with chronic diseases, as well as coordinating care with the psychosocial care network. In the context of a rapidly aging population, integrated strategies can help reduce psychological distress and improve quality of life.

Depression among older Brazilians was found to be associated with simultaneous presence of multiple chronic conditions, female sex, age and socioeconomic factors. Use of a latent dimension of comorbidities broadened the understanding of the interactions between chronic diseases and mental health, providing insights for planning public policies and care practices aimed at the elderly population.

## SUPPLEMENTARY MATERIAL

SUPPLEMENTARY MATERIAL

## Data Availability

The data used in this study are publicly available on the Brazilian Institute of Geography and Statistics website, via the 2019 National Health Survey: https://www.ibge.gov.br/estatisticas/sociais/saude/9160-pesquisa-nacional-de-saude.html?=&t=microdados. The processed data and analysis code may be made available by the authors upon request.
